# EMTReK Model for Advance Care Planning in Long-Term Care: Qualitative Findings from mySupport Study

**DOI:** 10.3390/geriatrics10060171

**Published:** 2025-12-18

**Authors:** Irene Hartigan, Catherine Buckley, Nicola Cornally, Kevin Brazil, Julie Doherty, Catherine Walshe, Andrew J. E. Harding, Nancy Preston, Laura Bavelaar, Jenny T. van der Steen, Paola Di Giulio, Silvia Gonella, Sharon Kaasalainen, Tamara Sussman, Bianca Tétrault, Martin Loučka, Karolína Vlčková, Rene A. Gonzales, on behalf of the mySupport Study Group

**Affiliations:** 1 School of Nursing and Midwifery, University College Cork, T12 AK54 Cork, Ireland; 2 Northridge House Education and Research Centre, St Luke’s Nursing Home, T12 H970 Cork, Ireland; 3 School of Nursing and Midwifery, Queen’s University Belfast, Belfast BT9 7BL, UK; 4 International Observatory on End-of-Life Care, Lancaster University, Lancaster LA1 4YW, UKa.harding5@lancaster.ac.uk (A.J.E.H.);; 5 Medical Research Ethics Committees United, St. Andonius Hospital, 3435 CM Nieuwegein, The Netherlands; 6 Department of Primary and Community Care, Radboudumc Alzheimer Center, Radboud University Medical Center, 6525 GA Nijmegen, The Netherlands; jtvandersteen@lumc.nl; 7 Department of Public Health and Primary Care, Leiden University Medical Center, Albnusdreef 2, 2333 ZG Leiden, The Netherlands; 8 Department of Public Health and Pediatrics, University of Torino, Via Santena, 5 Bis, 10126 Torino, Italy; 9 School of Nursing, McMaster University, Hamilton, ON L8P 0A1, Canada; 10 School of Social Work, McGill University, Montreal, QC H3A 1B9, Canada; 11 Third Faculty of Medicine, Charles University, Ruská 2411, 100 00 Praha, Czech Republic

**Keywords:** advance care planning, ACP, knowledge translation, communication, dissemination, research uptake

## Abstract

**Background/Objectives**: Conversations about end-of-life care or advance care planning are often difficult and emotionally challenging to initiate. Tailoring messages to the specific audiences can make these sensitive discussions more manageable and effective. The Evidence-based Model for the Transfer and Exchange of Research Knowledge (EMTReK), compromising six core components (message, stakeholders, processes, context, facilitation, and evaluation) offers a structured framework for research dissemination and knowledge transfer in palliative and long-term care settings. Knowledge translation bridges research and practice, with its effectiveness depending on stakeholder engagement, tailored communication, and systematic application of evidence in policy and practice. This study explores stakeholder perspectives on a dementia care intervention, using EMTReK as an analytical framework to examine how knowledge transfer and exchange (KTE) actions were implemented across long-term care settings. **Methods**: A qualitative analysis was conducted on primary data comprising case narratives from multinational research groups involved in the “Caregiver Decision Support” (mySupport) study (2019–2023). Teams from Canada, the Czech Republic, Ireland, Italy, the Netherlands, and the United Kingdom evaluated the mySupport intervention through interviews, with analysis guided by components of the EMTReK model. **Results**: Facilitated Family Care Conferences were found to be effective mechanisms for supporting knowledge transfer and intervention uptake in dementia care across nursing homes in Europe and Canada. Despite challenges posed by the COVID-19 pandemic, Family Care Conferences adapted through stakeholder engagement, interactive learning, and innovative communication methods. Using EMTReK as an analytical framework, the research team identified key elements that contributed to successful implementation, including the importance of flexibility to accommodate local contexts. **Conclusions**: The transnational application of the EMTReK model for advance care planning in long-term dementia care highlights the importance of tailored, culturally relevant knowledge translation strategies, which, despite challenges from the COVID-19 pandemic, were successfully implemented through local adaptations and diverse dissemination methods, emphasising the need for further research on their impact on resident and family outcomes.

## 1. Introduction

Knowledge translation describes several dynamic and iterative processes that bring together research and practice to provide more effective health services and strengthen the healthcare systems [1]. Knowledge translation strategies can optimise the uptake of knowledge and support the implementation of interventions into practice. Such strategies depend on understanding the target audience and the purpose of the translation, whether it is to inform or change clinical practice, behaviour, policy, organisations, or systems, and to influence research, academic, and educational communities [2].

The Family Carer Decision Support (FCDS) intervention is an educational programme designed to assist nursing home staff support family carers making end-of-life care decisions for relatives with advanced dementia. Initially implemented in 24 nursing homes in the United Kingdom, it reduced family carers’ uncertainty and improved perceptions of care quality [3]. The Family Carer Decision Support intervention was subsequently adapted for use in six countries; this process was described in detail by the transnational, multidisciplinary mySupport study [4] which employed knowledge translation strategies to promote understanding and uptake of end-of-life care and advance care planning in long-term care settings.

Across Europe, advance care planning is shaped by diverse cultural attitudes toward death and dying, which affect both knowledge and intervention uptake [5]. In some European countries, end-of-life discussions remain a taboo, hindering open advance care planning conversations between families and staff. Language barriers and cultural differences among both healthcare workers and family caregivers further complicate communication [6]. Variations in perspectives on palliative and end-of-life care highlight the need for culturally sensitive approaches to advance care planning, including tailored staff training and accessible information for families [7].

Applying knowledge transfer frameworks can also be challenging due to their abundance, which may cause confusion when choosing a suitable approach [8]. The most effective framework often depends on the specific context, sector, or challenge. Table 1 outlines commonly cited healthcare frameworks, which can be adapted to project goals. These models can be applied individually or in combination to leverage their strengths at different stages of the knowledge translation process [9].

EMTReK is used to ensure research knowledge is disseminated effectively within both the local and organisational context. Integrated knowledge translation involves en-gaging knowledge users from the outset as equal partners with researchers through the research process. By integrating these models, healthcare teams can tailor their knowledge transfer approaches to better address complex challenges and support the successful uptake of research into practice. The integration assumes that effective knowledge transfer and exchange is a prerequisite to successful implementation [10].

EMTReK identifies six key interdependent and equally critical components for effective KTE in palliative care [10].

The core components are as follows:

1. The message—the information or evidence to be communicated.
2. The process—the activities and methods enabling knowledge transfer, including facilitators and champions.
3. The stakeholders—the individuals or groups generating or applying knowledge [11]. These components operate within two types of contexts.
4. Local context—the immediate organisational setting (e.g., nursing homes).
5. Broader context—social, cultural, and economic factors shaping research and implementation.
6. Outcomes—mechanism to evaluate the success of the knowledge transfer.

**Figure 1:**
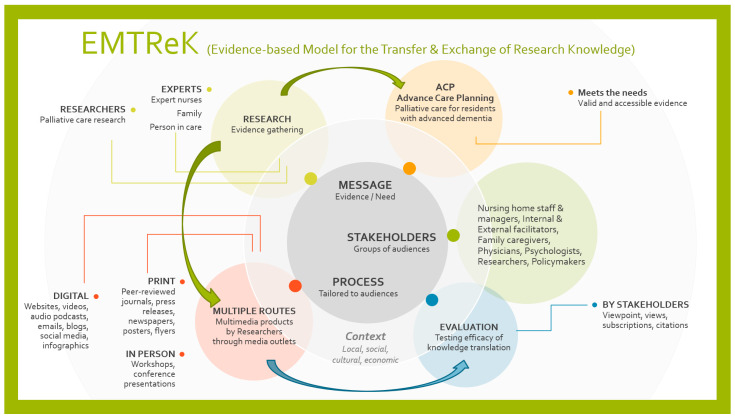
EMTReK diagram showing the components of Message, Stakeholders, and Process (Content: mySupport study; R Gonzales Designed in 2023).

Figure 1 illustrates the EMTReK model applied to the mySupport study, highlighting the dynamic interaction of components in practice. Its circular design emphasises the continuous nature of knowledge translation, where research shapes tailored messaging, disseminated through multiple routes (e.g., print, digital, in-person) and informed by stakeholders such as providers, family caregivers, researchers, and policymakers. Evaluation feeds back into the system to assess effectiveness and guide improvement, showing how knowledge is shared and meaningfully integrated into care practices while adapting to diverse organisational and cultural contexts across countries.

A critical component of EMTReK is evaluating efficacy, which involves assessing the success and impact of knowledge translation (KT) activities, ensuring that the knowledge shared leads to meaningful change in practice. In the context of palliative care for people with advanced dementia, EMTReK offers a valuable framework to analyse how multinational partners engage in KTE. For example, in the mySupport study, stakeholders such as internal and external facilitators, physicians, and family carers collaboratively influenced both the process and uptake of knowledge. The aim of this paper is to explore the experiences of nursing home staff, managers, family carers, and external facilitators in implementing a dementia care intervention across diverse settings. EMTReK was applied post hoc as an analytical framework to identify key elements of knowledge transfer and exchange (KTE) that influenced implementation.

An evaluation of the intervention’s application was conducted from the perspective of key stakeholders. In this study, stakeholders refer to representatives from each participating country, including researchers, senior academics, care home staff, family carers, and both internal and external facilitators. These individuals played a central role in adapting, implementing, and supporting the mySupport intervention within their local contexts.

A core component of the intervention, the Comfort Care Booklet, was designed to assist families and care teams in making informed decisions. It provides evidence-based guidance on when certain interventions may be beneficial and when they may not align with the goal of comfort-focused care [4,12,13]. 

Building on this foundation, the study explored the question, “What are stakeholders’ perspectives on the application of knowledge transfer and exchange (KTE) actions in the mySupport study?” Responses helped the research team identify key enablers, valuable elements, and aspects that were less effective or redundant, contributing to a deeper understanding of how KTE strategies functioned across diverse care settings.

## 2. Materials and Methods

Qualitative data from the international mySupport study (Advance Care Planning Intervention in Nursing Homes) explored knowledge transfer and exchange (KTE) for all consortium partners. Each partner country collected qualitative data through interviews with stakeholders, including family carers, nursing home staff, internal and external facilitators, and healthcare professionals. Interviews were conducted in the local language and transcribed verbatim. Country teams then compiled their findings into structured case narratives containing selected interview extracts and responses to a standardised template based on the EMTReK framework components (see Table 2), which were used as a scaffold to develop the interview questionnaires. For this study, these country case narratives served as the primary data sources for a cross-case analysis.

This study received ethical approval from multiple institutional and national ethics committees across participating countries. All participants were provided with detailed information regarding the nature and purpose of the research, their voluntary participation, and the handling of research data prior to giving informed consent. All procedures were conducted in accordance with the ethical standards of the relevant institutional and national research committees, and with the 1964 Helsinki declaration and its later amendments or comparable ethical standards. Development of interview guides, sampling procedures, and country-specific recruitment processes was aligned with COREQ guidance [14] for transparent reporting (items 9–13).

### 2.1. Patient and Public Involvement and Contribution

The Strategic Guiding Council, namely, patient and public involvement (PPI) and engagements, played a central role in tailoring the messages of the mySupport intervention to ensure relevance, clarity, and resonance for diverse audiences across participating countries. The council was composed of family carers and representatives from all partner countries, who were engaged at key junctures of the study and provided direct feedback on study materials and communication strategies, helping to shape how information was presented to both families and healthcare professionals [15].

Their involvement led to changes in the design and content of educational materials, emphasising the need to reach both the “hearts and minds” of families and staff, and ensuring that messages were culturally sensitive and accessible [16]. The council met regularly to review and discuss materials, offering insights based on lived experience, and supported translation and adaptation efforts to address local needs and contexts. Despite the COVID-19 pandemic, this collaborative approach not only improved the quality and translatability of the intervention but also fostered a more meaningful connection with stakeholders, ultimately enhancing the effectiveness of knowledge transfer and exchange within the mySupport study.

### 2.2. Introduction to the Advance Care Planning Intervention Study

The Advance Care Planning Intervention in Nursing Homes study was the main feature of the mySupport study and consisted of three components: (1) Training nursing home staff; (2) Provision of the Comfort Care Educational Booklet and question prompt list; and (3) The Family Care Conference where the internal facilitator meets family participants to discuss comfort care practices and preferences at the end of life in a one-hour Family Care Conference or meeting [3,17]. As reported by Brazil et al. in 2024 [17], in the mySupport study, nursing homes were enrolled from 6 countries: Canada, the Czech Republic, Italy, the Netherlands, the Republic of Ireland, and the United Kingdom. The RE-AIM framework (Reach, Effectiveness, Adoption, Implementation, and Maintenance) informed the development of case study templates and data collection tools. Data were collected in two phases (2020–2021) through semi-structured interviews conducted by master’s- or doctoral-level researchers [17]:

Phase 1: An environmental scan prior to the intervention, conducted with family carers, nursing aides, registered nurses, and nursing home managers, in which interviews examined attitudes, level of support, barriers to implementation, and potential cooperation related to the intervention.

**Figure 2:**
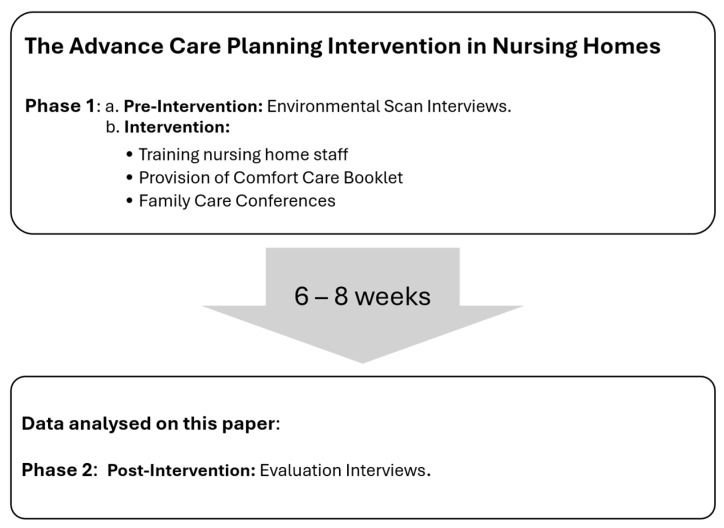
Phases of data collection of the Advanced Care Planning Intervention in Nursing Homes study. Researchers from the following participating countries submitted data for the mySupport study: Canada, the Czech Republic, Italy, the Netherlands, the Republic of Ireland, and the United Kingdom. This paper focuses on the analysis of data collected during Phase 2 (Content: mySupport study; R Gonzales Designed in 2025).

Phase 2: A post-intervention evaluation was conducted 6–8 weeks after the Family Care Conference with family carers, external and internal facilitators, nursing home managers, and healthcare professionals. The interviews investigated factors influencing intervention implementation, including its perceived usefulness, integration into resident care plans, impact on nursing home staff work experience, effects on nursing home operations, and family carer acceptability. The data collected during Phase 2 were analysed for this paper (see Figure 2).

### 2.3. Data Analysis:

The analysis followed the five-stage framework analysis method described by Ritchie and Spencer [18], consistent with COREQ [14] items 24–28 regarding systematic coding, matrix development, and verification of interpretations. Codebooks and individual-case nursing home templates were developed in an iterative process with researchers from each partner country. Framework analysis, based on the theoretical propositions described in the flagship paper [17], was applied to the cross-country analysis by experienced qualitative researchers located in each partner country (AC, EC, AH, LB, SG, KV, BT, JD). Researchers’ disciplinary backgrounds, qualitative experience, and prior relationships with sites were documented to enhance reflexivity (COREQ [14] items 1–8). Regular cross-country reflexive discussions were held to minimise bias and ensure analytic consistency. As part of the knowledge translation work package, this team met routinely online to review emerging themes, maintain coding consistency, resolve discrepancies, and refine analytic insights, thereby strengthening the validity and reliability of the findings across countries. 

The qualitative analysis drew on framework analysis, an approach suited to applied health research and multi-country policy-relevant studies [18]. This method enables structured comparison across cases and alignment with predetermined theoretical propositions. The components of the EMTReK model provided an additional scaffold that supported team-based, cross-national analysis and further strengthened analytic coherence. Using the framework approach [18], themes were charted as columns in a matrix, with data from each participating sites organised by country in the rows. Indexing involved systematically coding data segments and tagging them by country of origin. The indexed data were subsequently organized into a secondary matrix by targeted themes (e.g., Comfort Care Booklet, Family Care Conference). To avoid redundancy, overlapping statements were assigned sequential codes representing multiple sources.

For interpretation, we reviewed the chart to compare perceptions, accounts, and experiences, looking for patterns and connections to finally build a summary of the interviews using a narrative approach [19]. The EMTReK components were used as a scaffold for analysis, and indexes were retained to show the sources and to identify common views. The summary is available in the Appendix A and the findings are presented in the Section 3.

## 3. Results

Thirteen nursing homes across six countries completed the Advance Care Planning Intervention and participated in post-intervention interviews. A total of 296 interviews were conducted with key stakeholders, including family carers (n = 134), nursing home managers (n = 34), internal facilitators (n = 77), external facilitators (n = 11), nursing home staff (n = 28), and health professionals (n = 12) [20].

Interviews were distributed across two phases: Phase 1 (pre-intervention environmental scan) (n = 136) and Phase 2 (post-intervention evaluation) (n = 160). This paper focuses on the analysis of Phase 2 data, which explored stakeholder experiences and perceptions of the intervention. Thirteen researchers from six countries—Canada (n = 2), the Czech Republic (n = 2), Italy (n = 2), the Netherlands (n = 2), the Republic of Ireland (n = 2), and the United Kingdom (n = 3)—conducted narrative analyses using the EMTReK framework to assess knowledge transfer and exchange (KTE) actions within the mySupport study.

Country representatives examined how their interview findings aligned with the six components of KTE as outlined in EMTReK [11]. These findings contribute to a deeper understanding of how KTE strategies function across diverse long-term care environments and inform future scaling of Family Carer Decision Support interventions.

### 3.1. The Message

Regarding “the message”, researchers found that most home-care staff, managers, and family carers lacked an in-depth knowledge of advance care planning prior to the intervention. Those participants appreciated the booklet and workshops (Family Care Conferences) because they provided information and training on sensitive topics that are commonly avoided by family members. Experts from the mySupport study gathered and curated evidence as information on advance care planning for people with advanced dementia and made it available to champions, who participated in the translation to their local cultural environment, including language translation and adaptation to local practice and custom. The message, delivered in multiple formats, included tools to facilitate training, monitoring, and implementation of the study, tailored for local audiences in the participating countries. Researchers found that the translated material was actionable and recognised it as a useful resource that could spread within the caring community and to the public.

### 3.2. The Stakeholders

The stakeholders included a variety of audiences at multiple levels of the social structure in each country, from researchers to healthcare providers and managers, including family carers, training facilitators and academic institutions, persons living with dementia, and the public. The ultimate beneficiaries were family carers, nursing home healthcare professionals, residents in care, and palliative care researchers.

### 3.3. Multiple Processes

The processes used to implement knowledge transfer met the needs of the stakeholders, who discussed the adequacy of the information prior to the development of materials, considering the methods of communication available and used by each audience. Skilled external facilitators received support from researchers and trained internal facilitators and staff. Champions shared which type of marketing techniques helped promote the multiple activities and media products created to make knowledge accessible in the language of each champion. Although efficacy was not reported, the techniques included videos, audio podcasts, newsletters, flyers, informative emails, blogs and social media postings, informative websites, peer-reviewed journal publications, conference presentations, press releases for local newspapers, and content and training material for instructive workshops.

### 3.4. The Local Context

Local context consideration guided the association with local universities involved in research and training, with participation of nursing homes interested in development of their staff, facilitating training to improve their quality of care and their existing palliative care programmes. Researchers provided ongoing support and, when required, assisted in organising actions related to the study, establishing a partnership with nursing homes that helped resource the transfer of knowledge.

### 3.5. The Wider Social, Cultural and Economic Context

The social, cultural, and economic context dictated the adaptations required in the process of translation of knowledge, accommodating each participant country. Cultural differences were apparent with varying levels of acceptance of discussions about end-of-life care. In some countries, where the topic is culturally avoided, the approach required tact and consideration.

### 3.6. Evaluation of the Model

The *mySupport* study highlighted the importance of adapting Knowledge Transfer and Exchange (KTE) strategies to local cultural, organisational, and resource contexts. While the intervention demonstrated potential to inform future policy, generate cost savings, and optimise human resource allocation, the analysis went beyond a typical application of EMTReK. Rather than using EMTReK solely as a planning tool, we employed it post hoc as an analytical framework to deepen the interpretation of stakeholder experiences and uncover nuanced insights into implementation dynamics. Through this lens, key components of KTE such as credibility, accessibility, and relevance were visibly enacted across diverse long-term care settings. These elements emerged organically in the data, reflecting how stakeholders navigated implementation challenges and adapted strategies in real time. Details on the country of origin for the data referenced above are provided in the Appendix A. A thematic analysis of how core Knowledge Transfer and Exchange dimensions were reflected in the data is presented in Table 3. All settings demonstrated the presence of those core components, though operationalization varied according to stakeholder needs and infrastructural realities. Stakeholders including family carers, nursing home staff, managers, and researchers who participated in collaborative and reflective processes across countries.

Family Care Conferences, supported by culturally adapted training materials, served as a central mechanism for applying and sharing knowledge across diverse care settings. These conferences created structured opportunities for families, care staff, and facilitators to engage in meaningful conversations about values, preferences, and care goals for people living with dementia. The implementation of knowledge transfer was not a one-way process. Instead, it emerged as a dynamic “push–pull” interaction: researchers and facilitators actively introduced evidence-informed tools and approaches (the “push”), while care providers and families brought forward their own questions, needs, and contextual insights (the “pull”). This reciprocal exchange allowed for the co-construction of knowledge that was both relevant and responsive to local realities. By embedding these strategies into routine practice, the project ensured that knowledge was not only disseminated but also translated into action. This approach supported the successful uptake of the intervention across long-term care settings in six European countries and Canada, demonstrating the value of culturally sensitive, participatory methods in advancing person-centred dementia care.

The COVID-19 pandemic (2020–2022) significantly affected the study, presenting challenges to knowledge delivery and stakeholder engagement. However, it also prompt-ed the creative adoption of alternative communication methods, many of which were in-formed by patient and public involvement (PPI). Training shifted from in-person to online formats, and despite these obstacles, the dissemination of information to all stakeholders elicited positive responses. Participants anticipated ongoing improvements in the quality of care for individuals with advanced dementia, enhanced collaboration among nursing homes, family carers, and researchers, and greater integration of palliative care approaches to better address the complex needs at the end of life.

The findings underscore that while the overarching intervention message remained consistent, the operationalization of KTE components required flexibility and responsive-ness to context. For example, multimedia resources were broadly appreciated but used differently: digital platforms were preferred in Canada and the Netherlands, whereas print materials were more common in the Czech Republic. The credibility of knowledge shared was strengthened by the presence of skilled facilitators, the use of evidence-based materials, and the cultivation of trusted relationships. Notably, Family Care Conferences served as a central mechanism for applying knowledge in practice across all participating sites.

Stakeholder engagement and interactive learning emerged as pivotal, with co-creation contributing to intervention refinement. Facilitators played key roles in adapting content, navigating organisational readiness, and supporting sustainable change. Additionally, media-based dissemination strategies expanded the reach of the intervention beyond immediate care settings.

This cross-national implementation highlighted that while the core message of per-son-centred advance care planning is universal, the means of delivering and embedding it must be flexible. A tabulated summary of the thematic analysis of the application of EMTReK to the mySupport study is presented in Appendix A.

## 4. Discussion

This study used the EMTReK model as a post hoc analytical framework to examine the implementation of advance care planning in long-term care facilities across six countries, focusing on residents with advanced dementia and their families. While EMTReK was not applied directly by participants, its components provided a structured lens through which stakeholder experiences were interpreted. The findings demonstrate the framework’s utility in identifying key elements of Knowledge Transfer and Exchange (KTE) in long-term care environments, while also highlighting challenges and opportunities in transnational implementation. The use of a structured KTE framework supported consistent messaging across countries, while allowing for local variation in how knowledge was exchanged, acted upon, and sustained. This cross-national study illustrates that such frameworks can maintain a consistent focus on core principles while enabling culturally and organisationally appropriate adaptation, thereby enhancing the relevance and sustainability of person-centred dementia care interventions

### 4.1. Integration of EMTReK Components in Advance Care Planning Implementation

Our findings show that the six components of the EMTReK model provided a com-prehensive framework to facilitate the application of advance care planning interventions across diverse healthcare settings. Participants’ perspectives highlighted the interconnectedness of these components, particularly how message adaptation was influenced by local and broader sociocultural contexts. This finding aligns with previous research emphasising that effective knowledge translation requires tailoring to specific contexts while maintaining intervention fidelity [20]. Stakeholders’ recognition of the importance of culturally sensitive approaches to end-of-life discussions mirrors findings from Hanson et al. [21], who identified cultural adaptation as critical to successful advance care planning implementation in long-term care. The identification of knowledge gaps among healthcare staff and family caregivers regarding advance care planning is consistent with previous studies demonstrating limited understanding of advance care planning among nursing home staff and families of residents with dementia [3,17]. The structured approach of the EMTReK model helped fill these gaps by ensuring that educational materials and training were systematically delivered while accommodating local contexts. This targeted approach to knowledge dissemination appears particularly valuable in gerontological nursing, where evidence-based practice must be balanced with person-centred care principles.

### 4.2. Stakeholder Engagement and Interdisciplinary Collaboration

A significant strength of the EMTReK implementation was the engagement of diverse stakeholders across multiple levels. The involvement of researchers, healthcare providers, managers, family caregivers, and education institutions created a multifaceted support network for the intervention. This approach reflects recent shifts in implementation science toward collaborative, systems-based approaches rather than siloed interventions [22,23].

Similar stakeholder engagement was reported in studies (Archibald and O’Donnell) that emphasised the need for collaboration across disciplinary boundaries to effectively implement advance care planning interventions in nursing homes [22,23]. Their international, multiple-case study, like ours, found that engaging multiple stakeholders from the outset increased buy-in and fostered shared ownership of the intervention [23]. This collaborative approach appears particularly relevant for gerontological nursing, where complex care needs necessitate person-centred principles and interdisciplinary coordination [24].

Our findings regarding the value of external facilitators supported by researchers are noteworthy for nursing practice. Participants expressed that this structure provided necessary expertise while building internal capacity, suggesting a sustainable model for knowledge translation in long-term care settings. This aligns with previous research demonstrating that facilitation is a key component of successful implementation in nursing homes, particularly for complex interventions like advance care planning [25,26].

### 4.3. Adaptability and Resilience in Implementation

The COVID-19 pandemic presented significant challenges to implementation, yet champions demonstrated remarkable adaptability in delivering the intervention despite these obstacles. The pivot to alternative communication methods and creative approaches to training illustrates the resilience of the implementation process when guided by a structured model like EMTReK. This finding has important implications for nursing practice, suggesting that flexible, adaptable approaches to knowledge translation may be more sustainable in dynamic healthcare environments [27].

The staff’s ability to maintain intervention delivery during the pandemic also highlights the value of having a clearly articulated knowledge translation framework. While specific implementation strategies needed modification, the underlying EMTReK components provided stability and direction. This observation supports growing evidence that theory-based implementation frameworks improve intervention outcomes, particularly in complex settings like nursing homes [8].

### 4.4. Cultural and Contextual Considerations in Advance Care Planning Implementation

Our findings regarding cultural differences in acceptance of end-of-life discussions have significant implications for gerontological nursing practice. Participants shared varying levels of comfort with these discussions across countries, necessitating tactful adaptations while maintaining the core intervention elements. This cultural sensitivity is particularly important in dementia care, where family dynamics and cultural norms strongly influence decision-making processes [27].

O’Donnell et al. (2023) [23] similarly found that cultural context significantly impacted advance care planning implementation, with successful sites demonstrating careful attention to local norms and practices. Their work, like ours, underscores the importance of balancing intervention fidelity with cultural adaptability. For nursing practice, this suggests that advance care planning interventions must incorporate flexibility while maintaining core evidence-based components [23].

During the mySupport study, the champions dedicated time to translating materials to local languages and customs, which contributed to the practical application of contextual adaptation. This process went beyond simple translation to include consideration of terminology, approaches (such as blogs) to sensitive topics, and alignment with existing practices. Such thoughtful adaptation is essential for intervention acceptance and sustainability, particularly for interventions addressing sensitive end-of-life issues [8].

### 4.5. Implications for Gerontological Nursing Practice

The successful application of the EMTReK model has several important implications for gerontological nursing practice. First, it highlights the value of structured knowledge translation approaches for improving dementia care in long-term care settings. Nurses can serve as key stakeholders in implementing evidence-based interventions when provided with appropriate frameworks and support from researchers, champions and external facilitators.

Second, the emphasis on stakeholder engagement throughout the implementation process suggests that nurses should actively involve residents’ families and interdisciplinary team members when introducing new practices. This collaborative approach may increase acceptance and sustainability of practice changes [8,28].

Third, the findings regarding knowledge gaps among staff and family caregivers indicate a need for ongoing education about advance care planning in nursing homes. Gerontological nurses are well-positioned to lead educational initiatives like advance care planning, particularly when supported by evidence-based resources like those developed in this study [17].

Finally, the stakeholders’ perspectives on potential policy impacts suggest that nursing involvement in advance care planning implementation may contribute to broader system changes. By documenting outcomes and advocating for supportive policies, nurses can be empowered to translate successful interventions into sustainable practice improvements [29,30,31].

### 4.6. Limitations and Future Directions

Despite the valuable insights gained, several limitations must be acknowledged. First, the concurrent COVID-19 pandemic inevitably influenced the implementation process and may have shaped stakeholders’ perceptions of the intervention’s impact, particularly due to restrictions on in-person visits.

Second, while the cross-national design of the study is a strength in demonstrating the adaptability of the model, it also introduces complexity when comparing implementation across diverse healthcare systems. Future research could explore in greater depth how specific features of national healthcare systems influence the implementation of advance care planning interventions using the EMTReK framework [11].

Finally, this study focused on implementation processes and family carers’ perceptions. It did not assess patient clinical outcomes. Future studies should examine how EMTReK-guided implementations affect clinical outcomes, family satisfaction, and the alignment of care with residents’ preferences [9,11].

## 5. Conclusions

The transnational application of the EMTReK model to support advance care planning in long-term care settings demonstrates the value of structured knowledge translation approaches for improving dementia care. The mySupport study demonstrated that effective knowledge transfer and exchange in palliative dementia care requires tailoring messages to users’ needs, ensuring materials are culturally relevant, accessible, and grounded in both scientific and experiential knowledge. Despite challenges posed by the COVID-19 pandemic and cultural differences, champions successfully implemented the intervention by leveraging the components of EMTReK and adapting to local contexts. Local adaptation was essential: training was adjusted for language, digital capacity, and organisational structures. Skilled facilitators and internal champions played key roles in uptake, while organisational culture and leadership influenced readiness and resourcing. Dissemination leveraged both traditional academic channels and creative platforms such as podcasts, university networks, and social media to reach varied audiences. Future research should further examine the impact of such implementations on resident and family outcomes while continuing to refine knowledge translation approaches for complex nursing home environments.

## Figures and Tables

**Table 1:** Overview of frameworks and models that are commonly referenced in healthcare literature.

Framework/Model Name	Description	Typical Application in Healthcare
Knowledge to Action (KTA)	Overarching process from knowledge creation to implementation and sustainability	Guiding research into practice and policy
Evidence-based Model for the Transfer and Exchange of Research Knowledge (EMTReK)	Focuses on developing key messages for specific audiences and contexts	Research dissemination and stakeholder engagement
Practical Robust Implementation and Sustainability Model (PRISM)	Emphasises implementation, sustainability, and contextual fit of interventions	Sustaining evidence-based interventions
Diffusion of Innovations Theory	Describes how innovations diffuse across populations	Adoption of new practices or technologies
Understanding-User-Context Framework	Tailors’ knowledge translation by focusing on user needs and context	Customising knowledge translation (KT) strategies for different settings
Consolidated Framework for Implementation Research (CFIR)	Provides a comprehensive structure for assessing implementation across multiple domains	Implementation research and evaluation
Promoting Action on Research Implementation in Health Services (PARIHS)	Highlights the interplay between evidence, context, and facilitation in successful implementation	Facilitating evidence-based practice
Theoretical Domains Framework (TDF)	Identifies determinants of behaviour change	Designing behaviour change interventions
Ottawa Model of Research Use	Focuses on the process of research uptake and factors influencing use	Promoting research use in clinical practice
Knowledge Transfer Effectiveness (KTE)	Emphasises effectiveness of knowledge transfer processes	Technology transfer and innovation

**Table 2:** Components and subcomponents of EMTReK used as a scaffold to develop interview questionnaires.

Components and Subcomponents of Interview Questionnaires	
1. The Message	
Knowledge meets user’s need Knowledge is accessible Multiple types of knowledge are valid	Knowledge is credible Knowledge is actionable
2. The Stakeholders	
Involves multiple stakeholders Knowledge producers	Knowledge users (consumers) Knowledge beneficiaries
3. The Process	
Interactive exchange Skilled facilitation Opinion leaders/champions Marketing knowledge by knowledge being accessible Targeted, timely activities	Diverse activities Comfort Care Booklet (CCB) Family Care Conference Other conversations with staff Other media such as website, leaflets related to mySupport study
4. The Local Context	
Impact and influence of local setting on the transfer process Organisational influence Organisational culture	Readiness is key Resourcing KTE Social, cultural, and economic context Efficacy (evaluation)

**Table 3:** Summary of knowledge transfer and exchange actions ^1^ in the mySupport study.

KTE Component and Subcomponents	Summary of Insights from Participating Countries	Examples from Study Sites
The Message	Focused on person-centred advance care planning; adapted culturally through translated videos, booklets, and discussion guides.	All countries. Local relevance emphasised.
Knowledge Relevance	Addressed identified gaps in advance care planning knowledge; emphasised communication skills for end-of-life care planning.	Ireland, Netherlands, UK.
Knowledge Accessibility	Use of accessible formats (print, digital, video) to reach varied literacy and technological capacities.	Czech Republic (print); Canada and Netherlands (digital).
Credibility of Knowledge	Built through trusted facilitation, clinical documentation, and evidence-informed resources.	Noted in Italy, Ireland, and UK.
Multiple Knowledge Forms	Combined formal research, clinical experience, and carer perspectives; use of tablets and online platforms	Canada and Netherlands emphasised digital support.
Actionability	Training translated into practice through Family Care Conferences and local planning tools.	Italy and UK emphasised actionable outputs.
Stakeholders	Involved care staff, family carers, managers, students, and researchers in co-design and delivery.	Strong engagement in Ireland and UK.
Interactive Exchange	Supported through workshops, reflective practice, and informal dialogue, encouraging bidirectional learning.	Canada, Czech Republic, Ireland.
Facilitation and Leadership	Required proactive leadership and champions to support adaptation and implementation.	Strong facilitation in Canada and Netherlands.
Knowledge Dissemination	Strategies extended to podcasts, blogs, press releases, and social media to increase reach and impact.	Ireland and UK leveraged multiple media platforms.
Local Context	Delivery was influenced by language, staffing, cultural norms, and organisational structures.	Clear contrasts between Czech Republic and Canada.
Organisational Readiness and Resourcing	Contextual enablers included openness to innovation, leadership buy-in, and a culture of person-centred care. Practical supports (e.g., protected time, space, incentives) facilitated participation and sustainability.	Netherlands, UK, Ireland, and Italy highlighted resourcing needs.
Evaluating Efficacy	Palliative care interventions require comprehensive, multidimensional communication that reflects the complexity of patient and caregiver needs.	All countries valued the booklet as a resource to support continuity and coordination of care ^2^

^1^ Knowledge transfer and exchange actions refer to activities that promote the dissemination of information. ^2^ The Message, Stakeholders, and Local Context are processes that involve interactive knowledge exchange, skilled facilitation, strong leadership, tailored messaging, diverse activities, and timely adaptation. KTE: Knowledge Transfer and Exchange.

## Data Availability

Qualitative data sharing is not possible due to ethical and confidential concerns. This study was not preregistered.

## Abbreviations

The following abbreviations are used in this manuscript:

ACDs	Advance Care Directives
ACP	Advance Care Planning
CA	Canada
CCB	Comfort Care Booklet
CoP	Community of Practice
CZ	Czech Republic (Czechia)
EMTReK	Evidence-based Model for the Transfer and Exchange of Research Knowledge
FAMILY CARE CONFERENCE	Family Care Conference
FCDS	Family Carer Decision Support
iKT	Integrated Knowledge Translation
IRL	Republic of Ireland
IT	Italy
KT	Knowledge Translation
KTE	Knowledge Transfer and Exchange
NI	Northern Ireland
NL	The Netherlands
UK	United Kingdom

## Appendix A

Thematic summary of stakeholders’ application of EMTReK to mySupport study. The acronyms in parentheses indicate the country of origin of the preceding data, which corresponds to participating sites in the participating countries (see table legend).

**Table A1:** Summary of the evaluation of the application of EMTReK to the mySupport study by interview components and subcomponents.

Components /Subcomponents	Analysis of Application of Knowledge Transfer and Exchange (n = 160)
*The Message*	***Research produces knowledge. From this knowledge multiple messages can be derived.*** Researchers should reflect on the knowledge to be transferred and adapt their KTE plan accordingly. Includes components relating to the relevance, usability and quality of the knowledge to be transferred.
*Knowledge meets user’s need*	Most participants indicated that they did not have an in-depth knowledge of advance care planning prior to the intervention (IRL). In most cases, detailed resources specific to advance care planning were provided by the Family Care Conference and the Comfort Care Booklet, which enabled participants to explain to their families what the nursing home could do for their relative in care (IRL). **Nursing home staff** (CA, CZ, IT, NL, UK), **physicians, psychologists** (NL), **managers** (IT, NL), **internal facilitators** (IT), and **external facilitators** (CZ): Appreciated booklet and workshops for the information and training on discussing challenging topics (CZ, IT). Internal facilitators and family caregivers received written resources (IT). Some family members cited the sensitive topic as a reason why not to engage (UK). **Family caregivers** (CA, CZ, IT, NL, UK) informed at a Family Care Conference by external facilitator (CZ). **Researchers** shared that they hoped to be inspired to carry out similar projects in the future (CA, CZ, IT, NL). **Policymakers**: Were given information on the impact of family conferences on the quality of end-of-life care for nursing home residents and their family caregivers and potential cost savings. Evidence has potential for human resource allocation policies (IT). Or policymakers and other nursing home providers to be contacted in the future (CZ).
*Knowledge is accessible*	The booklet, question prompt list, and flyer were customised to suit language and practice in all partner countries (Czech, Dutch, English, French Canadian, and Italian) Arcand’s video had subtitles in all languages of participating partners. Infographic available in Italian. mySupport website available in Italian. Online training replicated in Czech by external facilitator. Developed training manual for staff in Czech. Online training offered in Canvas in three parts by external facilitator (CA) Environment: Training conducted during staff meetings online (accessed on site, from desk, or from home) (CA). International newsletters translated to Dutch. Information sheets in Dutch adhered to guidelines for human research. The UK partners had four online meetings with six external facilitators during three months of data collection, and there was a Community of Practice initiative lead by Queen’s University Belfast that was frustrated by events related to the COVID-19 pandemic. UK internal facilitators expressed that training could have been streamlined for clarity. Staff suggested adding an interactive element and an opportunity to ask questions and discuss issues. Study material for family carers could be more accessible.
*Multiple types of knowledge are valid*	Researchers collected data before and after interviews and after the conference (CZ). Clinical records and interviews were made available (CA, IT). The external facilitator research pack contained all the relevant documentation, including timesheets and reflection templates. Staff used individual Samsung tablets. Champions and Community of Practice (CoP) initiatives supported training and processing information (IRL). Researcher field notes kept (CA, CZ, IT) as “in-action” reflection (IT), while research diary (CA, IT) as “on-action” reflection (IT). These were used to develop new knowledge and strategies to implement the mySupport study (CA, IT). Study progress information and updates appeared in the newsletter including quantitative and qualitative data (NL). It was important to value all types of expertise: the external facilitator was an expert in advance care planning and dementia care, while the internal facilitators were experts in caring for their residents (UK)
*Knowledge is credible*	Credibility and trustworthiness achieved by consistent presence of the team on site and online, using reflexivity, debriefing, staff input, and collaboration. Use of an external facilitator to role model the intervention (CA). Knowledge originating from multiple sources, including interviews with staff and carers, facilitators, and client receipt inventory, is more credible and trustworthy. Data collected in the Czech Republic was analysed by more than one researcher. The external facilitator completed online training through Canvas and participated in an online Community of Practice in Ireland. Community of Practice online meetings were scheduled over two months with the participation of six external facilitators. Several debriefing sessions between facilitators and research team used for reflexivity, which was pivotal throughout the project (IT). Booklet developed by experts from several disciplines and intervention adopted by two experienced nurse practitioners in hospice and nursing home care (NL).
*Knowledge is actionable*	The intervention materials were tailored to local ethics and protocols. The booklet was based on best-practice approach to care and staff training matched their environment, hierarchical structures, and protocols (CA). Guidelines, podcasts, and blogs have potential to change practice provided the target audience is reached with all the produced knowledge (CZ). The family conferences (Family Carer Decision Support) were viewed as a means of clarifying information and coordinating communication with families; the Comfort Care Booklet was a useful resource to share with the family and a complement to the Family Care Conferences (IRL). The Family Carer Decision Support is seen as a positive addition to the Advance Care Directives used in the home. However, some families may be reluctant to engage in family meetings and discuss end-of-life issues (IRL). Staff suggested the Family Carer Decision Support may introduce discussions about Advance Care Directives to the family by providing information on the topic in a gentler manner (IRL). Tablets were supplied where needed to facilitate training. All internal facilitators were given manuals with information leaflets, a copy of the booklet, pre/post Family Care Conference questionnaires, PowerPoint slides with project timelines/schematic, timesheets, and researchers’ contact details (IRL). KTE activities, the developing of materials by participating countries, and data from the study were widespread on social media and other forums (IRL, IT). The booklet prompts family caregivers to discuss the information with relevant healthcare providers, including physicians, to draft a care plan. The training material includes implementation guidelines (NL). UK participants shared that the booklet could make a real difference to the lives of residents and family carers. The Family Care Conference provided additional structure for service delivery.
*The Stakeholders*	*Stakeholders include palliative care researchers, service users, carers—both informal and formal—and citizens with an interest in palliative care, palliative care service providers, academic institutions, health professionals—both specialist and generalist—and agencies.* Researchers should identify appropriate stakeholders to be involved in the transfer activities. This should include people on either and/or both sides of the exchange process.
*Involves multiple stakeholders*	Nursing home staff and managers, family caregivers, palliative care researchers (CA, CZ, IT, NL), physicians (CA, NL), head nurses, team leads, front-line care staff, social work interns (CA), academic institutions (CA, IT), persons living with dementia (CA), local health network of the region (CA), and potentially lay public through website and social media (CZ). UK suggested developing an easy-read knowledge transfer document targeted at decision makers.
*Knowledge producers*	From the perspective of knowledge producers, researchers coordinated the entire process from design to data collection and delivering workshops (CA, CZ, IT, NL). These workshops were held in person but matched the material available online in Canvas (CZ). Family caregivers and nursing home staff and managers also contributed to knowledge production (IT). Researchers capture the process using research diaries and in-the-field notes (IT). Field notes and decision-making templates were useful for documenting knowledge (UK). Both external and internal facilitators assisted with training (CA). Study progress continues to be shared with staff (CA).
*Knowledge users (consumers)*	From the perspective of knowledge users, nursing home staff and managers (CA, CZ, IT, NL, UK), physicians, nurses and front-line staff (CA), palliative care researchers and policymakers (CA, IT), and internal and external facilitators (CZ, UK) were involved.
*Knowledge beneficiaries*	From the perspective of knowledge beneficiaries, family caregivers (CA, CZ, IT, NL), residents with advanced dementia (CA, IT), nursing home healthcare professionals (CA, IT, UK), care partners (families and friends of people with dementia) (CA), and palliative care researchers and policymakers (CA) were involved.
*The Process*	From the perspective of the process, strategies for communication can include both traditional (peer-reviewed journal, abstracts, posters, speaking at conferences, workshops, etc.) and non-traditional tools (blogs, podcasts, open access web journals, infographics, leaflets) for knowledge dissemination. Researchers should identify appropriate processes or strategies for implementing the transfer. This is a “push–pull” process influenced by both the researchers’ actions and the needs of other stakeholders.
*Interactive exchange*	The in-person workshops gave the opportunity to use role-play to demonstrate the typical family conference and discussed the topic and booklet afterwards (CZ, NL, UK). An initial meeting with researchers, key informants, nursing home managers, and internal and external facilitators provided the setting to discuss the topic and materials (IT). Sharing new material, e.g., newsletters, videos, social media, was followed up by informal communication with the internal facilitator (CA, IT). Future updates by team leads planned (CA). Regular research meetings to discuss project issues (UK).
*Skilled facilitation*	In some instances, the external facilitator was a clinical social worker and a seasoned facilitator and programme developer with experience working in the local health system and caring for a person living with dementia (CA, NL), supported by research team and clinicians (CA). The researcher became the main contact for information for the nursing home and to provide psychological support (CZ, NL). Monthly meetings of researchers with internal and external facilitators used to discuss issues that needed solving in the project (IT).
*Opinion leaders/champions*	The nursing home manager was involved from the beginning and collaborated with internal facilitators (CZ). Key stakeholders included the following: General public, involved via outreach websites like FRidA (research forum lead by the university of Turin, Italy) that included links to promotion videos and to mySupport website, flyer, social media, infographic, press release, and dissemination through a local Alzheimer Café (IT). Educators, involved via UniTo (University of Turin) flash news with links to media mentioned above (IT). Students, involved via academic email and links to media above (IT). Nursing home staff involved by training workshops, external newsletters, and media above (IT). Training of internal facilitators and managers (CA). Internal facilitators helped in participant recruitment and promotion of the study in their areas during intervention (CA). Site managers made the study a priority during implementation (CA). A practical trainer or a quality nurse was involved in training (NL). Family caregivers involved via the comfort booklet, question prompt list, and media above (IT). Scientific societies involved via promotional video, flyer, and detailed study protocol aimed at Italian Societies of Geriatrics (IT). Policymakers and researchers involved via conference presentations and publications about mySupport study via peer-reviewed journals (IT). KTE champions involved via meetings to develop and enhance skills in KTE facilitation (UK).
*Marketing knowledge by knowledge being accessible*	**Video:** Promotional video of mySupport available in English with subtitles in all main languages spoken in the participating countries. Additional two-part training video for healthcare staff and care partners (CA, NI collaboration with Dr Arcand) (CA). mySupport video in English with French subtitles, shared by email on site (CA). **Audio:** Podcast recording with research assistance in 2021 for social work students (CA). Podcast recording with members of SGC about their involvement, experience, and impact (CA). **Digital publication:** Publication of mySupport study Newsletter [Tétrault, B. 2021] (CA). Information on mySupport study and copies of booklet and questionnaire shared with physicians and nurses and six care homes in the region (CA). Newsletter shared with nursing home managers and staff with regular updates on the project (IT). Italian flyer shared with nursing home staff and geriatric hospice societies (IT). Newsletter, intervention material, and email included the mySupport logo and layout (NL). Online package to launch in 2022: Initiative mySupport study, Creating Comfort Care Awareness (CA). UniTo Flash News newsletter circulated to approx. 3900 academic staff at the University of Turin including links to promotional video and mySupport website (IT). Academic informative email to approx. 81,000 students at the University of Turin including the above links (IT). mySupport informative package including links published on the FRidA (research forum lead by the university of Turin, Italy) website, with audience including researchers, university staff, students at university and high school, journalists, and lay population; the website counted 20,000 users in 2020 <https://frida.unito.it/57_my-support---un-supporto-per-le-scelte-difficili-di-fine-vita> (IT) (accessed on 4 May 2023). mySupport information package including links published on the homepage of the University of Turin’s new pages. **Print:** Press release in local newspaper covering nursing homes (IT). External facilitator created a brief overview of the Family Care Conference facilitator training (NL). Researcher created a factsheet with a scientific summary of the project in lay terms (NL). **Workshops:** Researchers and external facilitators provided workshops about the intervention for a broad audience (NL). **Translations:** Czech translation of all materials provided to nursing home staff, branded with mySupport study logo (CZ). Italian translation of all materials (IT).
*Diverse activities* - Comfort Care Booklet - Family Care Conference conversations with staff - Other media such as website and leaflets related to mySupport study	Dissemination of study materials, newsletter, weblinks, and flyers to staff, internal managers, participants, and other LTC homes (CA, IT, NL, UK). Informal phone calls, emails, in-person conversations, and factsheets (NL). Family Care Conference time slots tailored to staff availability (CA). The complete study presented in the Czech Journal *E-Psychology* and information available on the website: Centre for Palliative Care. (CZ). Nursing home managers and internal/external facilitators and the research team met at the start of the study to discuss the use of the booklet, this established trusting relationships (IT). Regular conversations between staff and researchers help define improvements in family recruitment (IT). One UK site made a small presentation to external colleagues to announce their involvement in the study, using videos, websites, and other resources.
*Targeted, timely activities*	From the perspective of targeted timely activities, regular checks were conducted to maintain momentum, and updates were provided periodically to managers (CA, CZ, IT, NL, UK). Regular email checks were sent to participants, with confirmations sent after completing tasks or attending events (CA). Contacts were intensified near recruitment and events (NL). All stakeholders asked to publicise the project and disseminate resources in a snowball approach (NL).
***The Local Context***	Researchers must consider the impact and influence that relevant local settings in which the transfer will occur can have on the process. Can include organisational settings.
*Impact and influence of local setting on the transfer process*	The organisation is part of the local health network that provides professional development opportunities for staff. The site is also linked to a university and is considered a research site. Efforts in place to share with regional health networks (CA). Staff and caregivers required translation and adaptation of all the material into Czech (CZ). Access to computers was limited, therefore requiring face-to-face training by an external facilitator (CZ, NL). Because care staff are not desk-based, printed information distributed to staff rooms might be more effective than online electronic information. This was a challenge in the context of remote delivery (UK).
*Organisational influence*	Managers and team lead promoted the involvement in professional development training in collaboration with researchers (CA, CZ, IT, NL). COVID-19 had a significant impact on the capacity to collaborate (CA). Internal facilitators felt supported by managers (IT).
*Organisational culture*	The culture of the organisation supported and welcomed professional development opportunities (CA). The organisation has experience hosting research studies on site (CA). The two participating nursing homes had different palliative care programmes in place; both benefitted from the Family Care Conferences (CZ). In one site, the manager appreciated the value of research in improving the quality of care provided for their residents; this appreciation was thought to be related to the higher education of that manager, which indirectly influenced the process of KTE (IT). Nursing homes are becoming more person-centred, recognising the importance of family carers and the need to inform them and get them more involved in the process of care (NL). Understanding an organisation’s values, priorities, strategies, and action plans can influence the application of KTE as an addition to the model they already have (UK).
*Readiness is key*	Sites had limited time for reflection and intervention and various levels of readiness for the study despite efforts from the research team, mainly because the study took place during the COVID-19 pandemic, which required prioritisation (CA). Nursing homes were prepared for change, having already started Family Care Conferences, and wanted to start with palliative care earlier (CZ). Nursing home staff and management were ready for change as they saw an opportunity to improve the quality of care and discuss neglected topics, education, and reflection. Internal facilitators looked forward to sustainable changes to overcome complacency and resistance to change (IT). In the second nursing home they were in strong need of change to improve communications among staff and managers and contact with family caregivers (NL).
*Resourcing KTE*	To help with implementation, uptake, and effectiveness, collaborations were built between researchers, care home staff, facilitators, and participants. Ongoing support provided to alleviate demand on care home, empower staff to engage in a new approach, and organise Family Care Conferences including room booking, invitations, and confirmations. Thank-you gift cards provided to internal facilitators afterwards (CA). Project activities were seen as a burden to nursing home staff and required some convincing (CZ). It was essential to establish a partnership with stakeholders to achieve an effective process of KTE (IT). Managers of the second nursing home facilitated staff extra hours to devote to the project (NL).
*Social, cultural and* *economic context*	The local culture considers talking about end-of-life and death as a taboo in Italy; family carers and nursing home managers avoid the terms “death” and “end of life”, and they are rarely discussed with families. This cultural context was considered in the translation of the materials given to family caregivers and written in lay terms for the public (IT). This is elaborated in a blog by the early career researchers available at https://mysupportstudy.eu/cultural-differences-and-advance-care-planning-for-residents-with-dementia-in-nursing-homes-emerging-considerations-and-recommendations-from-the-mysupport-study/ (accessed on 2 July 2022).
*Efficacy (evaluation)*	The COVID-19 pandemic caused a delay in the implementation of the study; staff had difficulty taking up the intervention, and the external facilitators provided additional requested training. Internal facilitators were supported and made the booklet accessible, but it was not utilised to its fullest potential. As internal facilitators expected, only one physician attended training despite all being invited. The booklet was considered clear and informative to all (CA). In the first nursing home, staff saw training as a burden and had no personnel to organise conferences; when these conferences occurred, no information was shared with those outside the participants. The second nursing home welcomed the study and will probably continue offering the Family Care Conferences. Outcomes of the project were shared with all staff (CZ). Dissemination strategies were tailored to stakeholders (IT).

The data in this table was reported by champions from the following participating countries: CA = Canada, CZ = Czech Republic, IRL = Ireland, IT = Italy, NL = The Netherlands, and UK = United Kingdom.
